# The interactions between major dietary patterns and rs320 polymorphism of LPL gene on cardiometabolic risk factors

**DOI:** 10.1038/s41598-025-27399-7

**Published:** 2025-12-10

**Authors:** Hooman Yekrang Safakar, Sayyed Saeed Khayyatzadeh, Zeinab Yazdanpanah, Mehdi Mollahosseini, Mohammad Hasan Sheikhha, Masoud Mirzaei, Hosein Fallahzadeh, Hassan Mozaffari-Khosravi

**Affiliations:** 1 Department of Nutrition, School of Public Health, Shahid Sadoughi University of Medical Sciences, Yazd, Iran; 2 Research Center for Food Hygiene and Safety, School of Public Health, Shahid Sadoughi University of Medical Sciences, Yazd, Iran; 3 Molecular and Medicine Research Center, Khomein University of Medical Sciences, Khomein, Iran; 4 Department of Genetics, Faculty of Medicine, Shahid Sadoughi University of Medical Sciences, Yazd, Iran; 5 Abortion Research Center, Yazd Reproductive Sciences Institute, Shahid Sadoughi University of Medical Sciences, Yazd, Iran; 6 Yazd Cardiovascular Research Center, Non-Communicable Diseases Research Institute, Shahid Sadoughi University of Medical Sciences, Yazd, Iran; 7 Departments of Biostatistics and Epidemiology, Research Center for Healthcare Data Modeling, School of Public Health, Shahid Sadoughi University of Medical Sciences, Yazd, Iran

**Keywords:** Lipoprotein lipase, rs320, Cardiometabolic, Dietary patterns, Polymorphism, Nutrition, Nutrigenomics, Predictive markers

## Abstract

There is a lack of comprehensive understanding concerning the variations in cardiometabolic parameters due to the interactions between dietary habits and Lipoprotein lipase (LPL) gene polymorphisms. This study aimed to investigate how primary dietary patterns relate to the Rs320 variant of the LPL gene and their impact on the cardiometabolic profile in a group of Iranian adults. This cross-sectional study involved 387 adults in Yazd, Iran, ranging in age from 20 to 70. Following an assessment of the inclusion and exclusion criteria, participants in the Yazd Health Study (YaHS) enrollment phase were chosen. In the present study, the major dietary patterns were identified using factor analysis method. The polymerase chain reaction-restricted fragment length polymorphism (PCR-RFLP) method was used to identify rs320 variant on LPL gene. General linear models (GLM) were applied to evaluate how dietary patterns interact with rs320 polymorphism to influence cardiometabolic markers. Three major dietary patterns were identified: western, healthy, and traditional. The allele distributions of rs320 were 66.5% for T and 33.5% for G. The prevalences of the genotypes were 57.90% (*n* = 224) for TT, 36.2% (*n* = 140) for TG, and 5.9% (*n* = 23) for GG. In unadjusted models, individuals with the TG + GG genotype exhibited higher odds ratios (ORs) for various factors, such as body mass index (BMI) exceeding 25 (OR: 1.36, 95% CI: 0.90–2.07), fasting blood sugar levels over 100 mg/dL (OR: 1.18, 95% CI: 0.74–1.87), and systolic blood pressure above 120 mmHg (OR: 1.41, 95% CI: 0.94–2.12). The interaction between traditional dietary patterns and the rs320 polymorphism was particularly significant. Total cholesterol and systolic blood pressure (SBP) revealed considerable crude p-values of 0.014 and 0.013, respectively. The adjusted models remained significance for SBP (*p* = 0.035). No cardiometabolic factors showed significant associations in the adjusted models for the western dietary pattern. Our findings suggest that extracted major dietary patterns are not associated with different genotypes of rs320 polymorphism in relation to the cardiometabolic profile. More extensive longitudinal research is required to elucidate the interaction.

**Supplementary Information:**

The online version contains supplementary material available at 10.1038/s41598-025-27399-7.

## Introduction

Cardiovascular diseases (CVDs) are the main cause of death globally, resulting in a substantial number of fatalities and disabilities. CVDs were accountable for 20.5 million deaths in 2021, representing around one-third of all deaths worldwide^1^. Although many major risk factors for cardiovascular disease are well established, some of the interactions and underlying mechanisms remain unclear. Some cardiometabolic risk markers, such as triglyceride (TG), high-serum total cholesterol (TC), high-density lipoprotein cholesterol (HDL-C), and low-density lipoprotein cholesterol (LDL-C), as well as hypertension and anthropometric measurements, have been found to potentially increase the risk of CVD development^2,3^. Diet plays a major role in CVDs, and dietary patterns such as a Mediterranean diet or Dietary Approaches to Stop Hypertension can alter the risk of these diseases^4^. It is widely accepted that the Western diets, characterized by high consumption of refined grains, red and processed meats, saturated fats, and high-fat dairy products, correlate with an increased risk of CVDs, while diets rich in fruits, fish, low-fat dairy, vegetables, whole grains, and nuts are associated with a reduced risk of CVDs and their contributing factors^5–7^.

Lipoprotein lipase (LPL) is identified as a key enzyme for lipid metabolism, and its abnormal levels are significantly associated with metabolic syndrome, diabetes, and cardiovascular diseases^8,9^. Triglycerides (TG) are hydrolyzed by LPL from circulating chylomicrons and very low-density lipoproteins (VLDL), resulting in the production of high-density lipoprotein (HDL-C) components and cholesterol-rich lipoprotein remnants. The LPL gene, located in region 8p22 and spanning approximately 30 kilobases (kb), encodes a 475 amino acid protein across nine of its ten exons^10^. Minimally, 88 different polymorphisms have been detected for the LPL gene across the human genome^11^. The HindIII (rs320) polymorphism, located at position 495 of intron 8, involves a substitution of G for T, modifying the enzyme’s recognition sites and reducing LPL activity^12^. Variations in the LPL gene might be linked to significant alteration in TG and HDL fractions^13^. Some differences in serum concentrations of lipid profiles might be explained by gene-diet interactions. Metabolic syndrome components were modified following interaction between PvuII and HindIII, two main LPL polymorphisms, with carbohydrate intake in the KMSRI-Seoul Study^14^. Recently, Faleti et al. highlighted the role of the rs320 polymorphism in lipid metabolism by stating that it had a significant correlation with triglyceride and cholesterol levels in a Nigerian population^15^. Additionally, Elsheimly et al. reported that LPL gene variants were related to a higher likelihood of type 2 diabetes and obesity in Egyptian adults. These findings indicate that LPL genetic variation may contribute to cardiometabolic risk, especially when interacting with environmental factors like diet^16^.

However, it remains to be elucidated whether different dietary patterns modify the cardiometabolic profile in the context of LPL genotype. Given that there are no LPL gene-diet interaction studies, to date, in Iranian populations, we examined the association of rs320 as one the most notable polymorphism of LPL and cardiometabolic risk markers in a sample of Iranian adults.

## Methods and materials

This cross-sectional study was conducted on a sub-sample of the Yazd Health Study (YaHS), including 387 healthy adults, aged 20 to 70, residing in the Greater Yazd Area located in central Iran. Participants were selected from the recruitment phase of an ongoing, population-based cohort study initiated in 2014, which includes 9,962 adults from the region. The YaHS, along with its subset study, Taghziyeh Mardom-e Yazd (TAMYZ), aims to investigate the incidence of non-communicable diseases and their potential risk factors. Details of these studies were published elsewhere^15,16^. The TAMYZ study has collected extensive nutritional data, including food frequency information. Since 2015, the Zist Bank-e-Yazd (ZIBA) has served as a biobank, preserving biological samples such as hair, nail, saliva, urine, serum, blood, and DNA for the YaHS study^15^.

To choose the participants, individuals from the YaHS study were selected according to inclusion criteria that included age between 20 and 70 years, availability of a whole blood sample in the YaHS biobank (ZIBA), and presence of nutritional and biochemical data in the YaHS and TAMYZ databases.

Individuals meeting any exclusion criteria, such as energy intake reporting anomalies (less than 800 or more than 4,200 kilocalories per day), susceptibility to allergic diseases, cancers, liver and kidney diseases, thyroid disorders, and acute or chronic infections, as per self-report, were excluded from the study. Also, use of medications influencing body composition, blood pressure, lipid levels, blood glucose, or mental health, such as glucocorticoids, statins, fibrates, cholestyramine, niacin, beta-blockers, renin-angiotensin system inhibitors (e.g., captopril, valsartan), insulin sensitizers (e.g., metformin), and insulin secretion stimulants (e.g., glibenclamide), and pregnancy or lactation status were excluded from the study.

### Dietary assessment

The dietary intake of participants in the TaMYZ study was assessed using a semi-quantitative food frequency questionnaire (FFQ) consisting of 178 items, which demonstrated adequate validity and reliability^16^. Participants provided information on their consumption frequency (times per month, week, or day) and portion sizes over the past 12 months. Portion sizes were estimated using a food photograph book, converted into standard units, and then expressed in grams per day. To facilitate dietary pattern analysis, food items were grouped into twenty-nine distinct categories.

To elucidate major dietary patterns, Principal Component Analysis (PCA) with varimax rotation was employed. This statistical technique serves to reduce complex datasets and extract dietary patterns by leveraging correlations among food groups. Initially, food groups were identified based on existing literature and then categorized accordingly. Subsequently, these groups were adjusted for individual energy intake using residual regression. The extensive list of food groups was then condensed into a smaller set of dietary patterns through factor analysis, guided by criteria such as scree plots and eigenvalues exceeding 1.5. To ensure data suitability and inter-variable relationships, the Kaiser-Meyer-Olkin measure and Bartlett’s test P-values were evaluated.

### Demographic and physical activity data

A standardized questionnaire given by trained interviewers was used to gather demographic data, such as age, sex, marital status, education level, employment position, smoking habits, and history of chronic diseases. The International Physical Activity Questionnaire (IPAQ) short form was used to measure physical activity levels, and the results were reported in metabolic equivalent minutes per week (MET-min/wk)^17^. Based on the median MET-h/wk values, participants were classified into sedentary, moderate, or active categories.

### Anthropometric and blood pressure measurements

A Bioimpedance Analyzer (Omron-BF511, Japan) was used to measure body weight, body fat percentage, and muscle mass percentage. A tape measure was used against a straight wall to measure height. Waist circumference (WC) and hip circumference (HC) were measured by trained interviewers. Body mass index (BMI) was calculated as weight (kg) divided by height (m²). The waist-to-hip ratio was derived by dividing WC by HC, with thresholds of ≥ 0.85 for women and ≥ 0.9 for men indicating abdominal obesity^18^. Blood pressure (systolic and diastolic) was measured after a 40-minute rest in a seated position, following two-thirds of the interview. Readings were taken three times at five-minute intervals, with the average of the second and third measurements used as the final blood pressure value. Measurements were conducted using Reichter electronic sphygmomanometers (Model N-Champion, Reister GMBH, Germany)^15^.

### Laboratory assessments

Biochemical markers, including TG, TC, LDL-C, HDL-C, and fasting blood glucose (FBG), were analyzed using commercial kits (Pars Azmoon) and calibrated autoanalyzers (Ciba Corning, Ciba Corp).

**Fig. 1:**
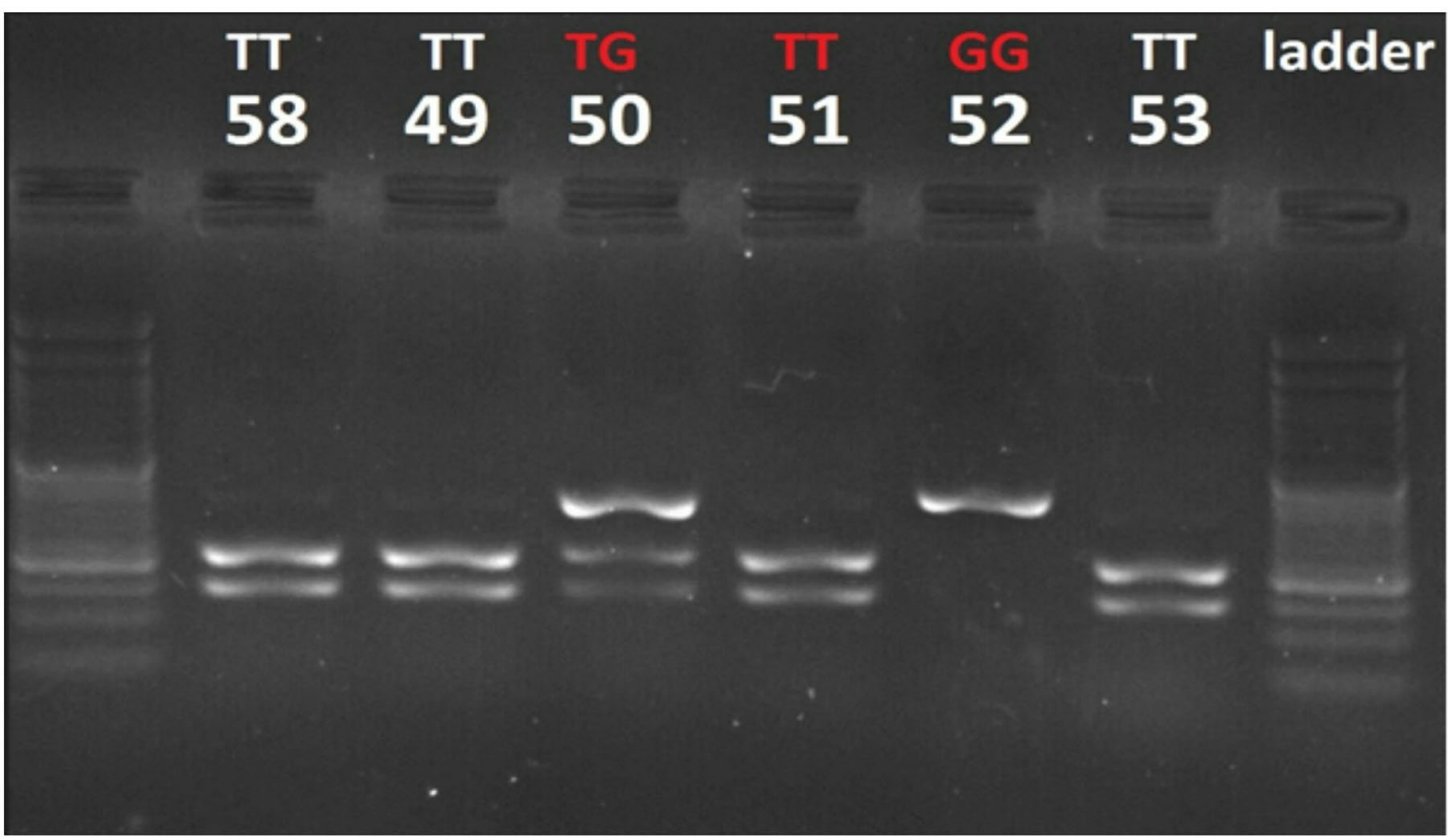
A stained agarose gel used to analyze rs320 polymorphisms of LPL gene. Larger DNA molecules are found towards the top of the gel and smaller DNA molecules are found towards the bottom of the gel. Lane right has a DNA ladder, and lanes 49 through 53 are samples from a restriction enzyme digestion of DNA molecule containing a single cut site. Sample GG contains uncut DNA, sample TG contains a mixture of uncut and cut DNA, and sample TT contains only cut DNA(Original gels are presented in Supplementary figure S1-S3).

A silica-based DNA extraction micro kit was used to extract genomic DNA from 300 µL of whole blood. Using the following primers, the rs320 (T >G) polymorphism was genotyped using polymerase chain reaction-restriction fragment length polymorphism (PCR-RFLP): forward 5′-GATGCTACCTGGATAATCAAAG − 3′ and reverse 5′-CTTCAGCTAGACATTGCTAGTGT − 3′. PCR reactions were carried out in a 25 µl volume, comprising 3 µl of extracted DNA, 1 µl of each primer (10 pmol/µl concentration), and 12.5 µl of Taq DNA Polymerase 2X master mix red. The amplification protocol included an initial denaturation at 95℃ for 10 min, followed by 38 cycles of denaturation at 95℃, annealing at 56℃, and extension at 72℃ (30 s each), concluding with a final extension at 72℃ for 5 min.A 0.5 µl aliquot of restriction enzyme (Thermo Fisher Scientific, USA) digested the 10 µl PCR products for four hours at 37 °C. The LPL-*Hin*dIII genotypes were determined according to Anderson et al.^17^. Following electrophoresis in a 3.5% agarose gel, the digested DNA fragments (10 µL) were seen. Three potential genotypes were identified: GG (138 and 217 bp), TG (217, 138, and 355 bp), and TT (355 bp) (Fig. 1). By sequencing randomly chosen samples using an ABI3130XL genetic analyzer from Applied Biosystems, the precision of the PCR-RFLP results was confirmed.

### Statistical analysis

We assumed a minor allele frequency (MAF) of G at 0.2616. A total of 387 eligible participants were included in the study. The Kolmogorov-Smirnov test assessed the normal distribution of variables. Genotype frequencies for the single nucleotide polymorphism (SNP) were evaluated for Hardy-Weinberg equilibrium using Pearson’s χ2 test, and non-quantitative data were analyzed similarly. Principal component analysis (PCA) identified dietary patterns from twenty-nine food groups. Continuous variables were compared using one-way ANOVA with the Bonferroni post-hoc test. The general linear model (GLM) and logistic regression were used to evaluate the impact of dietary patterns and the rs320 polymorphism on quantitative and qualitative variables, respectively, before and after adjusting for confounders like age, sex, education, marital status, physical activity energy intake, and occupation.

Participants were categorized into TT, TG, and GG genotypes, following previous studies^6,7,10^. The TT genotype was selected as the reference group, as it represents the wild-type allele and was the most frequent genotype. IBM SPSS version 25.0 conducted the statistical analysis. P-values ≤ 0.05 indicated statistical significance, while values less than 0.1 were considered marginally significant for interactions.

## Results

Table 1 summarizes the characteristics of the participants. Among the 387 healthy participants in the study were 194 women (50.1%) and 193 males (49.9%), all between the ages of 20 and 70. The BMI and weight mean ± SD were 26.51 ± 4.92 kg/m^2^ and 71.72 ± 14.26 kg, respectively. The distribution of rs320’s T and G alleles was 75.3% and 24.7%, respectively. The percentages of genotypes were 57.90% (*n* = 224) for TT, 36.2% (*n* = 140) for TG, and 5.9% (*n* = 23) for GG, and these results matched what is expected according to Hardy-Weinberg equilibrium (*P* = 0.52). No significant differences were observed in general, anthropometric, or biochemical parameters across genotype groups except for total cholesterol and diastolic blood pressure (*P* > 0.05, Table 1).

**Table 1:** Characteristics of the study participant across rs320genotypes.

Variables		Total (*n* = 387)	TT (*n* = 224)	TG/GG (*n* = 163)	*p*-value
Age; n (%)	20–29	86 (22.23)	52 (23.2)	34 (20.9)	0.94
	30–39	106 (27.40)	61 (27.2)	45 (27.6)	
	40–49	95 (24.54)	52 (23.2)	43 (26.4)	
	50–59	67 (17.31)	40 (17.9)	27 (16.6)	
	60–69	33 (8.52)	19 (8.5)	14 (8.6)	
Sex (%)	Male	193 (49.9)	118 (52.7)	75 (46.0)	0.19
	Female	194 (50.1)	106 (47.3)	88 (54.0)	
Weight (kg)		71.72 ± 14.26	71.34 ± 13.70	72.24 ± 15.02	0.54
BMI (kg/m^2^)		26.34 ± 4.66	26.06 ± 4.38	26.73 ± 5.02	0.16
WC (cm)		91.60 ± 12.16	90.82 ± 12.04	92.67 ± 12.27	0.14
Hip circumference (cm)		101.01 ± 11.89	100.44 ± 11.93	101.78 ± 11.83	0.27
WHR		0.89 ± 0.07	0.89 ± 0.07	0.90 ± 0.08	0.08
WHtR		0.56 ± 0.08	0.55 ± 0.07	0.56 ± 0.08	0.053
FBG (mg/dl)		95.08 ± 10.02	94.42 ± 9.85	96.01 ± 10.21	0.13
TC (mg/dl)		189.59 ± 36.99	186.21 ± 35.00	194.17 ± 39.18	0.03
HDL-C (mg/dl)		47.17 ± 9.66	46.72 ± 10.14	47.78 ± 8.97	0.29
LDL-C (mg/dl)		113.86 ± 32.34	111.91 ± 30.85	116.56 ± 34.22	0.16
TG (mg/dl)		130.10 ± 57.68	131.64 ± 57.55	127.93 ± 57.97	0.54
SBP (mmHg)		122.41 ± 13.21	121.43 ± 13.21	123.74 ± 13.13	0.09
DBP (mmHg)		77.80 ± 10.22	76.78 ± 10.41	79.19 ± 9.81	0.02

. ^1^ The values are given as mean ± standard deviation (SD), unless otherwise noted. Chi-square analysis and Independent Student’s t-tests used for categorical and continuous variables, respectively.

SD: standard deviation; TG: triglyceride; BMI: body mass index; WC: waist circumference; WHR: waist-to- hip ratio; LDL-C: low-density lipoprotein; TC: total cholesterol; HDL-C: high-density lipoprotein; SBP: systolic blood pressure; FBG: fasting blood glucose; DBP: diastolic blood pressure.

Three main eating patterns were discovered using principal component analysis: The Western Dietary Pattern (WDP) is defined by a diet high in red and processed meats, soft drinks, condiments, snacks, mayonnaise, pizza and sweets and low in grains; the Healthy Dietary Pattern (HDP) is characterized by a diet high in fruits, seafood, vegetables, and dairy products; and the Traditional Dietary Pattern (TDP) is characterized by a diet high in tea, salt, eggs, tomatoes, and grains and low in pizza. These three patterns accounted for 24.82% of the total variance in dietary intake. No significant associations were found between WDP and the body composition and biochemical parameters. However, in the crude model, significant associations were observed across HDP tertiles for body muscle percentage (*P* = 0.04) and HDL cholesterol (*P* = 0.02). These associations became non-significant (*P* ≥ 0.05) after adjusting for age, sex, energy intake, physical activity, occupational status, education level, and marital status.

### Multi-variable adjusted odds ratios for cardiovascular disease risk factors across rs320 genotypes

The analysis of cardiovascular disease risk factors across rs320 genotypes revealed non-significant trends. In crude models, individuals with the TG + GG genotype demonstrated higher odds ratios (ORs) for several factors, including a BMI > 25 (OR: 1.36, 95% CI: 0.90–2.07), fasting blood sugar > 100 mg/dL (OR: 1.18, 95% CI: 0.74–1.87), and systolic blood pressure > 120 mmHg (OR: 1.41, 95% CI: 0.94–2.12). Adjusted models, adjusted for age, sex, physical activity, energy intake, education, marital status, and occupation, exhibited marginal differences, with TG + GG carriers showing increased but statistically non-significant ORs for these parameters compared to the TT reference genotype. Overall, both crude and adjusted models demonstrated non-significant ORs for cardiometabolic factors among TG + GG carriers (Table 2).

**Table 2:** Multi-variable adjusted odds ratios (ORs) for risk factor of cardiovascular disease across genotypes rs320.

Variables	Models	Aalleles	OR	95% CI	*P*‑value
BMI > 25(kg/m^2^)	Crude	TT		References	0.13
	Adjusted	TG + GG TT	1.36	0.90 to 2.07 References	0.47
FBS > 100(mg/dl)	Crude	TG + GG TT	1.18	0.74 to 1.87 References	0.12
		TG + GG	1.40	0.90 to 2.17	
	Adjusted	TT		References	0.22
		TG + GG	1.34	0.83 to 2.15	
SBP > 12(mmHg)	Crude	TT		References	0.09
		TG + GG	1.41	0.94 to 2.12	
	Adjusted	TT		References	0.06
DBP > 80(mmHg)	Crude	TG + GG TT	1.54	0.98 to 2.41 References	0.10
	Adjusted	TG + GG TT	1.40	0.93 to 2.13 References	0.11
TC > 200(mg/dl)	Crude	TG + GG TT	1.44	0.91 to 2.27 References	0.17
		TG + GG	1.33	0.87 to 2.02	
	Adjusted	TT		References	
HDL-C < 40(mg/dl)	Crude	TG + GG TT	1.25	0.79 to 1.98 References	0.32 0.058
	Adjusted	TG + GG TT	0.63	0.39 to 1.01 References	
TG > 150(mg/dl)	Crude	TG + GG TT	0.70	0.41 to 1.17 References	0.17 0.83
	Adjusted	TG + GG TT	0.95	0.62 to 1.45 References	
Gallagher Classification	Crude	TG + GG TT	0.84	0.53 to 1.33 References	0.46 0.71
	Adjusted	TG + GG TT	0.92	0.61 to 1.39 References	0.28
LDL > 100(mg/dl)		TG + GG	0.78	0.50 to 1.22	
	Crude	TT		References	0.53
	Adjusted	TG + GG TT	1.14	0.74 to 1.74 References	0.97
		TG + GG	1.009	0.63 to 1.60	

The interaction between dietary patterns and rs320 genotypes on cardiometabolic factors was also investigated. The GLM model explored the interaction of food intake patterns and rs320 polymorphism on quantitative variables, with P-Interaction values for each variable and food pattern provided in Tables 3, 4 and 5. Adherence to food patterns was categorized into three levels, and rs320 genotypes were divided into two groups for analysis.

**Table 3:** Interaction between healthy food pattern and rs320 polymorphism and interaction between healthy food pattern and rs320 polymorphism adjusted for age, sex, energy intake, physical activity, occupational, educational and marital status.

Cardiometabolic factors	*P* value	*P* value adjusted
Weight (Kg)	0.502	0.631
BMI (kg/m^2^)	0.418	0.490
Waist circumference (cm)	0.352	0.504
Hip circumference (cm)	0.474	0.516
Fat percentage (%)	0.853	0.506
FBG (mg/dl)	0.116	0.169
HDL (mg/dl)	**0.035**	0.160
LDL (mg/dl)	0.373	0.290
TG (mg/dl)	0.446	0.270
Total cholestrol (mg/dl)	0.172	0.145
SBP (mmHg)	0.560	0.557
DBP (mmHg)	**0.028**	**0.061**
WHtR	0.335	0.360
WHR	0.210	0.232

*P*-value < 0.05 considers as statistical significant.

**Table 4:** Interaction between Western food pattern and rs320 polymorphism and interaction between Western food pattern and rs320 polymorphism adjusted for age, sex, energy intake, physical activity, occupational, educational and marital status.

Cardiometabolic factors	*P* value	*P* value/adjusted
Weight (Kg)	**0.079**	0.226
BMI (kg/m^2^)	0.144	0.381
Waist circumference (cm)	**0.080**	0.196
Hip circumference (cm)	0.330	0.442
Fat percentage (%)	0.444	0.789
FBG (mg/dl)	0.691	0.419
HDL (mg/dl)	0.613	0.991
LDL (mg/dl)	0.249	0.691
TG (mg/dl)	0.160	0.354
Total cholestrol (mg/dl)	**0.014**	0.153
SBP (mmHg)	**0.013**	**0.035**
DBP (mmHg)	**0.050**	**0.077**
WHtR	0.156	0.465
WHR	**0.073**	**0.073**

*P*-value < 0.05 considers as statistical significant.

Significant interactions between a healthy dietary pattern and rs320 polymorphism were not observed for most cardiometabolic factors. However, HDL levels (*p* = 0.035, unadjusted) and diastolic blood pressure (DBP) (*p* = 0.028, unadjusted) showed some association, though these effects were diminished after adjusting for age, sex, and lifestyle variables (*p* = 0.160 and *p* = 0.061, respectively) (Table 3).

**Fig. 2:**
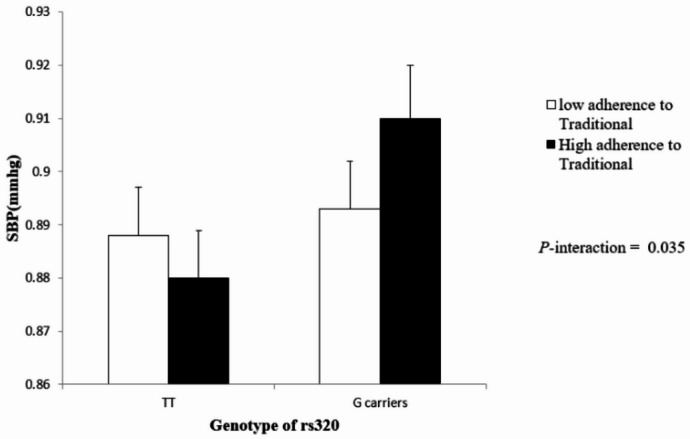
The interaction between the rs320 polymorphism of LPL gene and Traditional diet on systolic blood pressure (SBP); Measures are adjusted for age, sex, energy intake, physical activity, socioeconomic status and education level.

A statistically significant interaction was observed between the traditional dietary pattern and rs320 polymorphisms. Total cholesterol and systolic blood pressure (SBP) showed significant crude p-values of 0.014 and 0.013, respectively. Adjusted models retained significance for SBP (*p* = 0.035), suggesting a potential influence of traditional dietary habits on blood pressure regulation in rs320 polymorphism carriers (Fig. 2). Other factors, such as BMI, waist circumference, and DBP, exhibited borderline significance in crude models, but this did not persist after adjustment (Table 4).

**Table 5:** Interaction between traditional food pattern and rs320 polymorphism and interaction between traditional food pattern and rs320 polymorphism adjusted for age, sex, energy intake, physical activity, occupational, educational and marital status.

Cardiometabolic factors	*P* value	*P* value/adjusted
Weight (Kg)	0.404	0.565
BMI (kg/m^2^)	0.301	0.847
Waist circumference (cm)	0.521	0.767
Hip circumference (cm)	**0.090**	0.202
Fat percentage (%)	0.590	0.987
FBG (mg/dl)	0.613	0.464
HDL (mg/dl)	0.693	0.404
LDL (mg/dl)	0.414	0.736
TG (mg/dl)	0.286	0.450
Total cholestrol (mg/dl)	**0.064**	0.368
SBP (mmHg)	0.228	0.283
DBP (mmHg)	0.114	**0.077**
WHtR	0.106	0.617
WHR	**0.064**	**0.094**

*P*-value < 0.05 considers as statistical significant.

The Western dietary pattern showed limited interaction with rs320 polymorphism. None of the cardiometabolic factors demonstrated significant associations in adjusted models, with crude p-values for parameters like total cholesterol (*p* = 0.064) and waist-to-hip ratio (WHR) (*p* = 0.064) nearing significance. However, these effects diminished after adjustment, suggesting a limited interaction between Western dietary practices and the rs320 polymorphism (Table 5). These results might have significant clinical implications. High blood pressure and total cholesterol are two of the main preventable risk factors for cardiovascular disease. Those with the G allele may react more strongly to traditional dietary elements, including foods high in carbohydrates or salt, which are common in the traditional Iranian diet, considering that gene-diet interaction affects these characteristics. Therefore, modifying dietary recommendations in accordance with genetic predispositions may be a useful tactic to lower cardiovascular risk in people who are predisposed to it.

Analysis of the general linear model was conducted on both the male and female groups and the results are as follows:

HDP was not associated with any factors in the male group. On the other hand, after adjusting the cofounders, a significant interaction on TG and a marginally significant interaction on HDL have been shown in the female group. Significant interaction between TDP and genotypes was demonstrated on systolic blood pressure in man, and it remained the same after adjusting the cofounders. The female group had different results compared to men, and a significant interaction was observed in both the crude and adjusted models on total cholesterol. Although a significant interaction is demonstrated on waist-to-hip ratio, this interaction becomes marginal after adjustment. A marginally significant interaction between WDP and genotypes on systolic blood pressure and also a significant interaction on FBG were observed in the male group after adjustment. Finally, analysis in the female group showed a marginally significant interaction between WDP and genotypes on FBG and diastolic blood pressure and a significant interaction on TG in both the crude and adjusted models.

## Discussion

This study examined the association between the rs320 polymorphism, related to lipoprotein lipase levels, and cardiovascular disease risk factors, as well as potential interactions between this polymorphism and prevalent dietary patterns on cardiometabolic risk factors. The study involved participants from the adult population aged 20–70 in Yazd city during the initial stage (registration and sampling).

The three dietary patterns identified in this study appear to reflect the economic status and nutritional knowledge of the participants. No statistically significant associations were found between dietary patterns and socioeconomic factors. The Western dietary pattern is typically adopted by individuals with higher economic means but lower nutritional awareness. This group tends to consume less of the cheaper staples like grains and more of the costlier items such as red and processed meats and restaurant food. Conversely, the healthy dietary pattern is associated with individuals who have both the financial resources and the nutritional knowledge to choose beneficial yet more expensive foods, including fish, fruits, and dairy products. The traditional pattern seems prevalent among those with lower economic status, who primarily consume home-cooked meals (evidenced by low pizza consumption) and rely on more affordable food sources like grains and eggs. These results are in line with earlier research on the relationship between rs320 and cardiometabolic risk.

### Genetic associations and study findings

The findings of this study provide new insights into the interaction between the rs320 polymorphism of the LPL gene and dietary patterns in relation to cardiometabolic risk factors. In this study, we found that the rs320 genotype altered the relationship between food patterns and cardiometabolic indicators, especially for systolic blood pressure (SBP). Compared to TT carriers, those with the G allele (TG + GG) who followed a traditional food pattern had significantly greater SBP. The direction and strength of these interactions hint at a possible gene–diet modulation impact, even if certain relationships (such as those with HDL-C and total cholesterol) did not remain statistically significant after correction. While our study did not find a direct association between rs320 polymorphism and cardiometabolic parameters, significant and marginal interactions were observed when dietary patterns were considered. These results are partially consistent with, but also diverge from, previous studies investigating the role of LPL polymorphisms in lipid metabolism and cardiovascular health.

Previous studies have demonstrated a potential association between the LPL rs320 polymorphism and lipid metabolism, particularly in relation to total cholesterol, triglycerides, and HDL levels. Some studies have linked the G allele with an increased risk of metabolic disorders. For instance, a study by Munshi et al. in an Indian population found no association between rs320 and ischemic stroke, suggesting that this polymorphism may not have a universal effect on cardiovascular disease risk^12^. Similarly, our study did not find a direct association between rs320 and fasting blood glucose, triglycerides, or LDL-C levels.

Conversely, studies conducted in Russia and Saudi Arabia have reported that the G allele variant of rs320 is associated with increased total cholesterol, LDL-C, and triglycerides, particularly in individuals with a predisposition to type 2 diabetes mellitus (T2DM)^18,19^. These findings align with the results of Kochetova et al., who reported a significant relationship between the G allele and elevated cholesterol levels in individuals with normal weight^19^. However, in our study, the association between rs320 and total cholesterol was only significant in the context of the traditional dietary pattern, highlighting the importance of gene-diet interactions in modulating lipid metabolism.Furthermore, a study by Bogari et al. in a Saudi Arabian population reported a significant association between the rs320 polymorphism and higher levels of total cholesterol, LDL-C, and triglycerides in coronary artery disease (CAD) patients^18^. While our findings do not support a direct effect of rs320 on lipid parameters, the interaction analyses suggest that dietary patterns may modify the impact of this polymorphism on cardiovascular risk factors.

The gene-diet interaction hypothesis suggests that genetic variations in lipid metabolism genes, such as LPL, can influence individual responses to dietary intake. Several studies have explored interactions between LPL polymorphisms and macronutrient intake, providing mixed results.A study by Kim et al. (2013) in South Korea demonstrated that metabolic syndrome components were significantly modified by interactions between LPL polymorphisms (including rs320) and carbohydrate intake^14^.Similar lipid changes were noted by Faleti et al. (2023) in a study involving a Nigerian population, with the rs320 polymorphism demonstrating a strong connection to higher TG and TC levels^15^. Elsheimly et al. (2021) found a strong correlation between LPL gene variations, such as rs320, and a higher risk of obesity and type 2 diabetes in an Egyptian population. Their research supports the idea that rs320 may contribute to cardiometabolic dysregulation through a variety of mechanisms, highlighting the wider significance of LPL polymorphisms in affecting metabolic health^16^. Additionally, the KMSRI-Seoul Study found that individuals carrying specific LPL variants exhibited altered lipid profiles depending on carbohydrate intake^14^. This supports our finding that adherence to a traditional dietary pattern, which is carbohydrate-rich, may influence lipid parameters differently in individuals with different rs320 genotypes. These results from several populations highlight how rs320 regulates lipid metabolism. In addition to lipid profiles, this finding supports the notion that dietary composition plays a critical role in determining the metabolic effects of LPL genetic variations. In our study, we identified significant interactions between the traditional dietary pattern and the rs320 polymorphism on total cholesterol and systolic blood pressure, which aligns with previous research indicating that high-carbohydrate diets may differentially affect lipid metabolism in individuals with specific LPL genotypes. Those with genetically decreased LPL activity may see comparable modifying effects from our traditional dietary pattern, which is generally heavy in salt and refined carbs.

The LPL enzyme’s function may be affected by the rs320 polymorphism, which is located in intron 8 of the gene and is believed to affect mRNA stability or splicing. In order to create HDL, LPL is essential for converting triglycerides from chylomicrons and very-low-density lipoproteins (VLDL). Decreased LPL activity, especially in those with the G gene, can lead to lipid buildup, increased TG, and consequent effects on blood pressure and insulin resistance, especially when combined with diets high in carbohydrates.

The exact way in which the rs320 SNP influences dietary patterns remains largely unclear. Potential biological mechanisms underlying the interaction between rs320 polymorphism and dietary patterns may involve LPL enzyme activity and lipid metabolism regulation. LPL plays a crucial role in triglyceride hydrolysis, and the rs320 polymorphism has been associated with alterations in LPL activity^20^. Our study found significant interactions between rs320 and dietary patterns on HDL cholesterol, supporting the hypothesis that dietary composition may modulate the effects of LPL genetic variants on lipid fractions. These patterns suggest a clinically meaningful and biologically tenable pathway that may facilitate precision nutrition approaches, especially in populations that take a traditional diet.

### Limitations and strengths

This study’s strengths include the use of a validated FFQ^21^ administered by trained interviewers and the adjustment for potential confounding factors such as age, gender, energy intake, physical activity, education, and marital status. To our knowledge, this is the first study to explore the interaction between rs320 polymorphism and dietary habits on cardiometabolic disease risk factors.

However, the study is not without limitations. The FFQ method is susceptible to recall bias, and health status and disease information were self-reported, potentially leading to selection bias. Causality cannot be inferred because the study design was cross-sectional.

## Conclusion

This study revealed that the interaction between the rs320 polymorphism of the LPL gene and dietary patterns, particularly the traditional dietary pattern, may influence specific cardiometabolic risk factors such as systolic blood pressure. Considering that gene-diet interactions may play a significant role in metabolic health, these findings highlight the importance of considering gene-diet interactions in public health nutrition and cardiovascular prevention strategies such as targeted dietary interventions. Future cohort and RCT studies are warranted to explore these interactions further.

## Supplementary Information

Below is the link to the electronic supplementary material.

Supplementary Material 1

## Data Availability

Data set generated during the present study will be available from the corresponding author upon reasonable request.
