# Supplementary material for: The interactions between major dietary patterns and rs320 polymorphism of LPL gene on cardiometabolic risk factors
Source: Sci Rep. 2025 Dec 10;15:43549. doi: 10.1038/s41598-025-27399-7 (PMC12696105; doi:10.1038/s41598-025-27399-7)
Supplement: Supplementary file 1 — Supplementary Material 1 [file 41598_2025_27399_MOESM1_ESM.pdf]

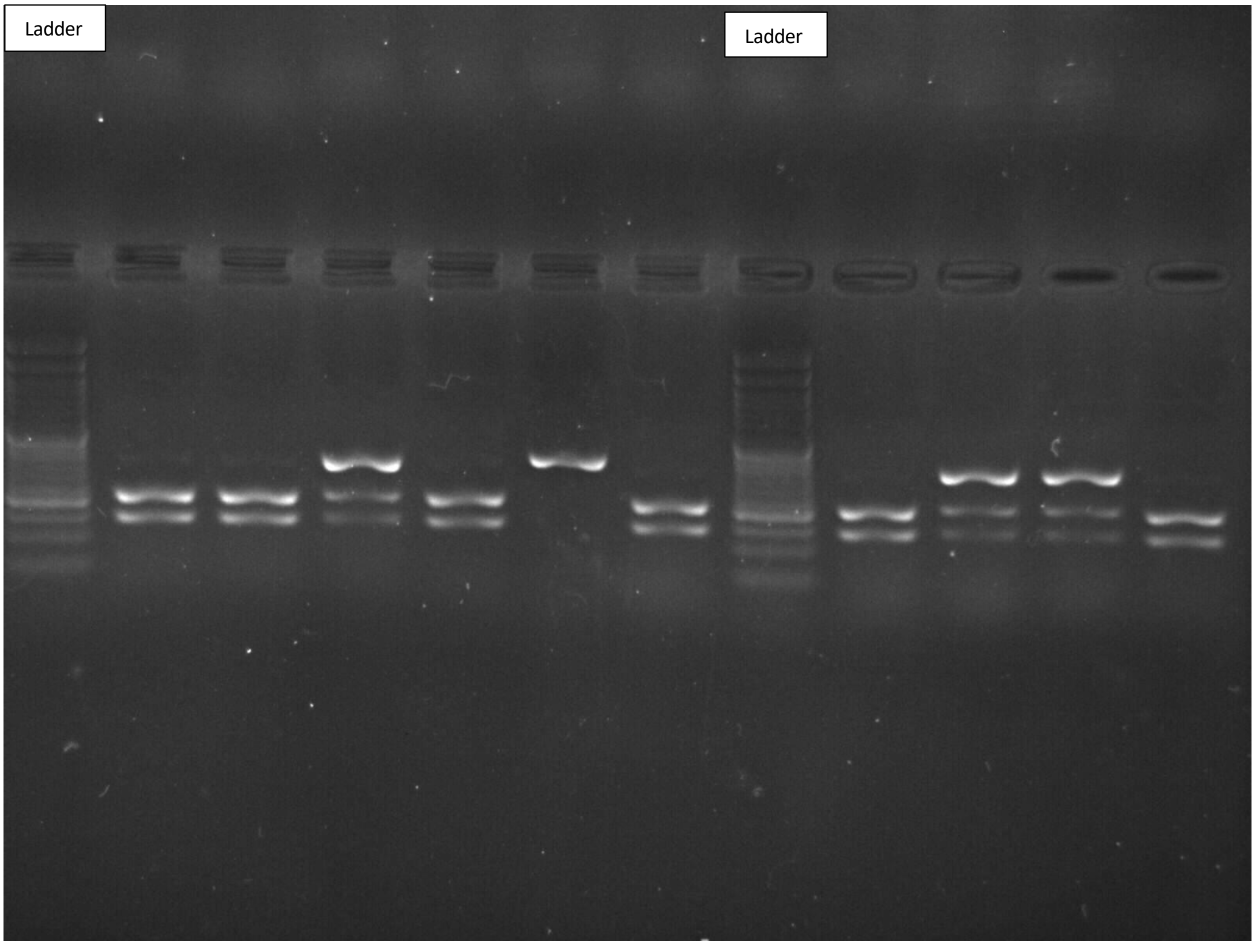


S1-A stained agarose gel used to analyze rs320 polymorphisms of LPL gene. Larger DNA molecules are found towards the top of the gel and smaller DNA molecules are found towards the bottom of the gel. Lane Left and lane 5 from right has a DNA ladder, and other lanes are samples from a restriction enzyme digestion of DNA molecule containing a single cut site. Sample with single DNA molecule lane in top contains only the G allele and is uncut DNA, samples with 2 parallel DNA molecules below contains only the T allele and is cut DNA, sample with 3 parallel DNA molecules contains both T and G alleles and it is a mixture of uncut and cut DNA.

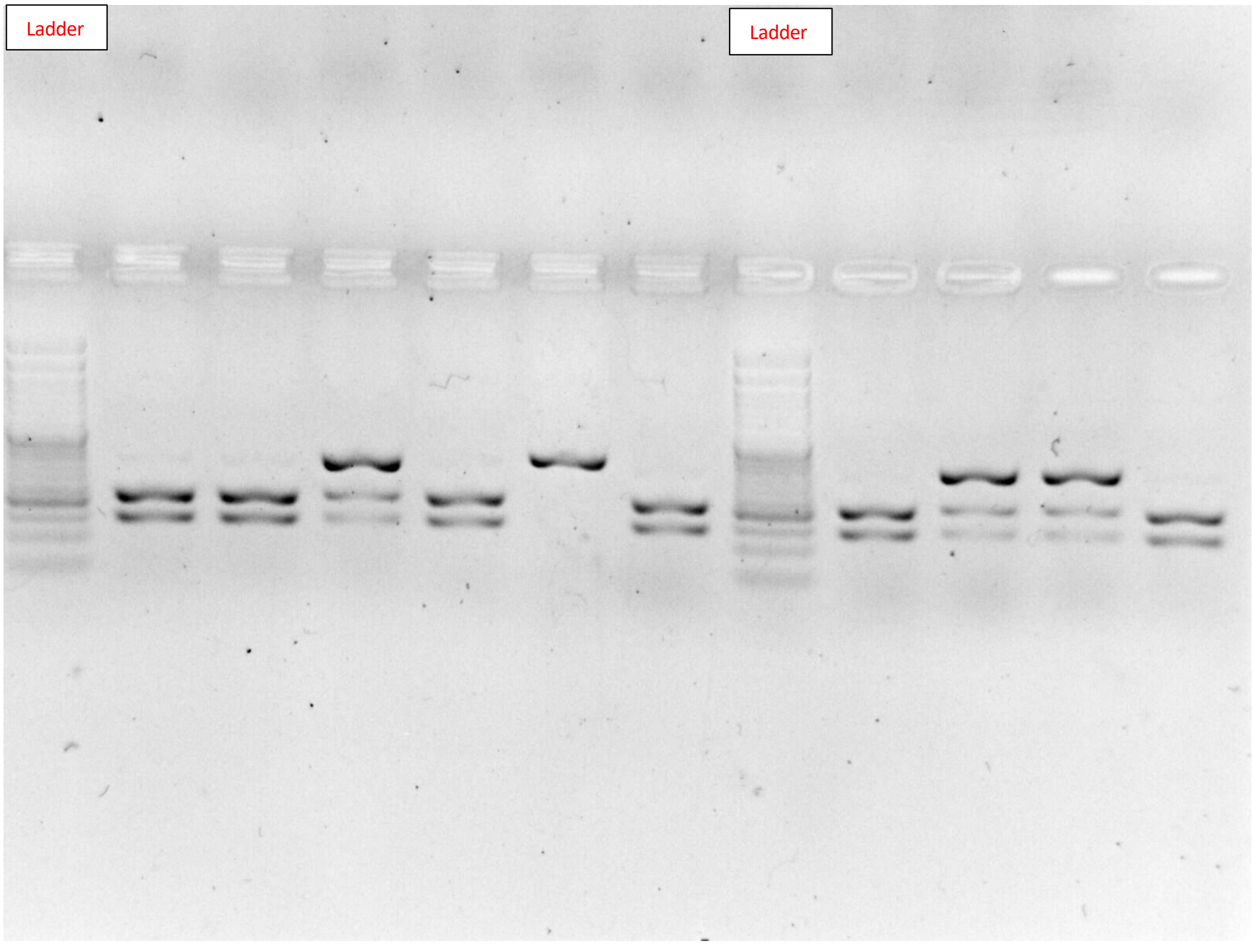


S2- A stained agarose gel used to analyze rs320 polymorphisms of LPL gene. Larger DNA molecules are found towards the top of the gel and smaller DNA molecules are found towards the bottom of the gel. Lane Left and lane 5 from right has a DNA ladder, and other lanes are samples from a restriction enzyme digestion of DNA molecule containing a single cut site. Sample with single DNA molecule lane in top contains only the G allele and is uncut DNA, samples with 2 parallel DNA molecules below contains only the T allele and is cut DNA, sample with 3 parallel DNA molecules contains both T and G alleles and it is a mixture of uncut and cut DNA.

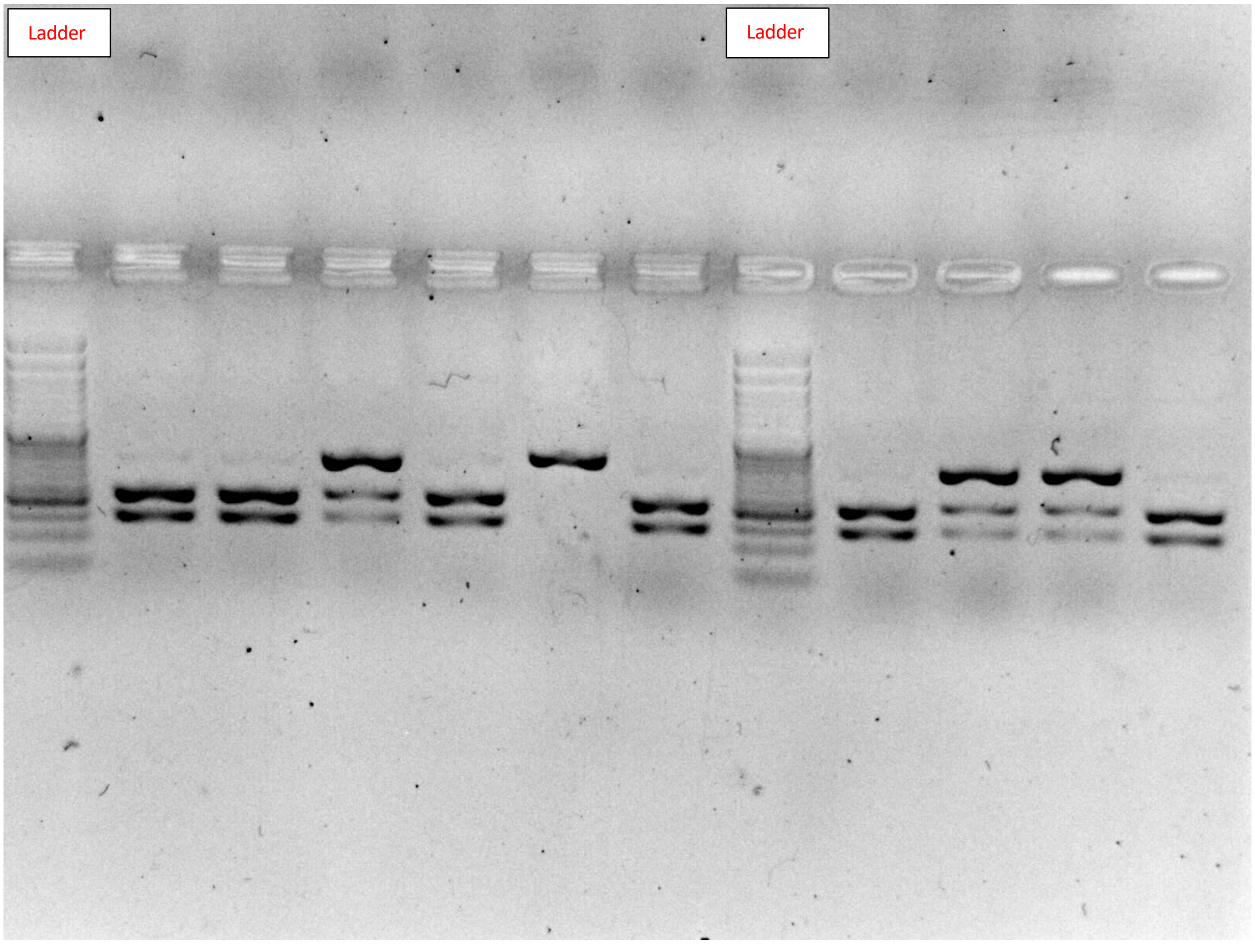


S3- A stained agarose gel used to analyze rs320 polymorphisms of LPL gene. Larger DNA molecules are found towards the top of the gel and smaller DNA molecules are found towards the bottom of the gel. Lane Left and lane 5 from right has a DNA ladder, and other lanes are samples from a restriction enzyme digestion of DNA molecule containing a single cut site. Sample with single DNA molecule lane in top contains only the G allele and is uncut DNA, samples with 2 parallel DNA molecules below contains only the T allele and is cut DNA, sample with 3 parallel DNA molecules contains both T and G alleles and it is a mixture of uncut and cut DNA.
